# Supplementary material for: A logistic regression model based on inpatient health records to predict drug-induced liver injury caused by ramipril—An angiotensin-converting enzyme inhibitor
Source: PLoS One. 2022 Aug 17;17(8):e0272786. doi: 10.1371/journal.pone.0272786 (PMC9384991; doi:10.1371/journal.pone.0272786)
Supplement: Attachment — Submitted filename: Response to Reviewer.docx [file pone.0272786.s002.docx]

Dear editor and reviewers,

Thank you for giving us the opportunity to submit a revised draft of the manuscript “**A logistic regression model based on inpatient health records to predict drug-induced liver injury caused by ramipril – an angiotensin-converting enzyme inhibitor**” for publication in *PLOS ONE*. We appreciate the time and effort that you and the reviewers dedicated to providing feedback on our manuscript and are grateful for the insightful comments on and valuable improvements to our paper. We have incorporated most of the suggestions made by the reviewers. Please see below, editor/reviewer comments are black and our responses are in blue.

**Editor comments**

1. Please ensure that your manuscript meets PLOS ONE's style requirements, including those for file naming.

Thank you for your comment. We have prepared our files according to the PLOS ONE’s requirements

2. Please provide additional details regarding participant consent.

Thank you for your suggestion. The Ethical considerations was revised as below:

The study protocols were reviewed and approved by the Institutional Review Board (IRB) of Hai Phong International Hospital, Vietnam (IRB. 20.318). The study was conducted in accordance with the Declaration of Helsinki and the International Conference on the Harmonization of the Technical Requirements for the Registration of Pharmaceuticals for Human Use - Good Clinical Practice guidelines. Prior to data collection, ethical approval was obtained from the ethics subcommittees of Hai Phong International Hospital. All subjects provided written informed consent prior to the commencement of the study.

3. In your Data Availability statement, you have not specified where the minimal data set underlying the results described in your manuscript can be found. PLOS defines a study's minimal data set as the underlying data used to reach the conclusions drawn in the manuscript and any additional data required to replicate the reported study findings in their entirety. All PLOS journals require that the minimal data set be made fully available.

Thank you for your comment.

 We uploaded our study’s minimal underlying data set as a Supporting Information file

**Reviewer #1: Reviewer comments**

i. In line 25 in the abstract there is an error

Thank you for your comment. We revised our manuscript based on your suggestion.

ii. In line 40, there is a statement that “age has been associated with a higher risk of Drug Induced Liver Injury” but it is not clear the direction of association.

Thank you for your comment. We have edited and clarified the relation between age and DILI in the introduction

iv. In line 41, the authors have indicated that “female sex appear to be more susceptible to DILI compared to man” Authors should consider using a more appropriate adjective “male”

Thank you for your comment. We revised our manuscript based on your suggestion.
v. In line 44, the authors indicated the National Institutes of Health maintains a searchable database of drug but there is no indication where this institute is. This should be made clear.

Thank you for your comment. We revised our manuscript based on your suggestion.
vi. The authors assessed the risk factors of DILI during ramipril and establish a logistic regression model to estimate the DILI of inpatients using ramipril in Vietnamese people. How the authors did insured that all the participants in this study were Vietnamese since they were using retrospective hospital records? What was the definition of someone to be Vietnamese in this study?

Thanks for your question. At the patient information system, clinicians (doctors, nurses) collected information about the patient's race. Thanks to that, we can easily identify the population of the research subjects who are Vietnamese

vii. The authors have indicated that the used data for the year 2018 to 2020. What was the justification for using data between this periods? What was the defined follow-up time in this study since it is a retrospective cohort study?

Thank you for your question.

The follow-up period of the study was 1 year from the date of DILI detection. For instance, patients diagnosed with DILI in 2019 were followed up until 2019. Similarly, those who were first diagnosed with DILI in 2019 were followed up until 2020.

This explanation has been added to the manuscript

viii. In this study there is no indication when it was conducted.

Thank you for your suggestion. We revised our manuscript as below:

Our study conducted in 2021 including data collected from the electronic medical records of inpatients from 2018 to 2020 at Hai Phong International General Hospital.

ix. In line 73, authors have indicated that this study was reviewed and approved by the Hai Phong International Hospital Institutional Review Board, Vietnam but there is no reference to provide evidence.

Thank you for your suggestion. Research review process by the hospital's IRB committee according to the procedures of the Ministry of Health of Vietnam. The approval of the Board is in official writing, however it is written in Vietnamese. We are ready to provide if the Review Board and the Editorial Board request

x. In line 74, there is a statement that this study was conducted in accordance with the Declaration of Helsinki and the International Conference on the Harmonization of the Technical Requirements for the Registration of Pharmaceuticals for Human Use - Good Clinical Practice guidelines. This deserves to be referenced.

Thank you for your suggestion. The Ethical considerations was revised as below:

The study protocols were reviewed and approved by the Hai Phong International Hospital Institutional Review Board, Vietnam. The study was conducted in accordance with the Declaration of Helsinki and the International Conference on the Harmonization of the Technical Requirements for the Registration of Pharmaceuticals for Human Use - Good Clinical Practice guidelines. Prior to data collection, ethical approval was obtained from the ethics subcommittees of the Hai Phong International Hospital. All subjects gave written informed consent before study initiation. If the subject is a child, a consent letter to participate in the study was obtained from the guardian

xi. Even if this data was obtained from some database, include epidemiological questionnaire that was also used to gather sociodemographic and clinical data for this study. Please explain thoroughly how the questionnaire was designed, implemented, filled, and checked for accuracy and completeness.

Thank you for your comment. RUCAM is the main tool for us to evaluate the causality if a DILI is suspect. This is a scale that has been developed, evaluated and widely used in the US, Europe and the world since 1989 (1) (2). We added the follow paragraph on Methods:

DILI was detected according to the Roussel Uclaf Causality Assessment Method (RUCAM) scales containing time to onset, course of ALT after taking drugs, alcohol use, age, concomitant drugs, previous drug hepatotoxicity, and response to unintentional drug re-exposure. For each criterion, the widest scale ranged from -3 to +3 with 7 degrees (-3, -2, -1, 0, +1, +2, +3), corresponding to the increasing probability of the role of the evaluated drug. The total score was classified into 5 degrees: ≤ 0, excluded; 1–2, unlikely; 3–5, possible; 6–8, probable; and ≥9, highly probable (3)

xii. In line 78, what ACEB compounds?

Thank you for your comments. We added the required data as below:

All patients in ramipril group were administered ramipril at a dose of 2.5 mg or 5 mg. The other ACEIs that the patients took were enalapril (2.5 mg/5 mg/10 mg/20 mg), perindopril (5 mg), lisinopril (5 mg/10 mg/20 mg), and captopril (12,5 mg/25 mg/50 mg).

xiii. In line 82, there is an indication that ALT and ALP had upper normal limit. What were these limits?

Thank you for your comments. We added information in Methods as below:

The ULN of ALT was 40 U/L, while that of ALP was 140 U/L.

xiv. Authors need to indicate the duration of exposure to ramipril and dosage as well as those on ACEIs.

Thank you for your comment. We added the data as your suggestion into Table 2. The data interpretation was added in the results

The duration of ramipril use in the ramipril group was similar to that of other ACEIs

xv. Since this study used clinical assessment as part of DILI diagnosis and it is possible that different physicians with different clinical experience could have led to similar patients classified differently or different patients classified in the same group resulting in misclassification. How did authors dealt with this issue?

Thanks for your comment. Clinicians diagnose DILI according to the RUCAM scale. This is supplemented and explained in the methods section. This scale helps different doctors to diagnose DILI equally.

xvi. Specifically, what clinical parameters were used for DILI diagnosis by clinicians?

DILI was detected according to the Roussel Uclaf Causality Assessment Method (RUCAM) scales containing time to onset, course of ALT after taking drugs, alcohol use, age, concomitant drugs, previous drug hepatotoxicity, and response to unintentional drug re-exposure. For each criterion, the widest scale ranged from -3 to +3 with 7 degrees (-3, -2, -1, 0, +1, +2, +3), corresponding to the increasing probability of the role of the evaluated drug. The total score was classified into 5 degrees: ≤ 0, excluded; 1–2, unlikely; 3–5, possible; 6–8, probable; and ≥9, highly probable (3)

xvii. It is important for authors to report the other drugs patients were on apart from ramipril since this not a randomized clinical trial. Further, dosages and duration of treatment for ramipril and ACEIs that were used should be reported.

Thank you for your comment. We added the drug study section that described more detail as follow:

**Drug study:**

All patients in ramipril group were administered ramipril at a dose of 2.5 mg or 5 mg. The other ACEIs that the patients took were enalapril (2.5 mg/5 mg/10 mg/20 mg), perindopril (5 mg), lisinopril (5 mg/10 mg/20 mg), and captopril (12,5 mg/25 mg/50 mg).

xviii. In line 100, the authors have indicated that they collected data on height, weight and BMI. BMI can be important in explain DILI but this variable and others has not be reported anywhere in the results section. What could be the possible explanation for this omission?

BMIs of the two groups were similar and did not contribute statistically to the development of the constructed model. Therefore, we did not include BMI in the model building variables. However, to be more explicit about the clinical indicators we collected, we have added BMI data for both groups to Table 2.

xix. In line 101 one of the variable collected was history but it is not clear. History about what?

Thank you for your comment. We have revised our manuscript as request

xx. In line 106 authors have indicated that they mapped the variables collected by the MHR to 32 discrete variables and they have referred to Figure 2 which is not detailed. It is not clear what these 32 variables were?

Thank you for your comment. We made the mistake of forgetting to cite figure 2. I added figure 2 to account for the variables evaluated during modeling.

xxi. In line 111, R statistical software should include manufacturer, name and country should be added in the manuscript.

Thank you for your comment. We have revised our manuscript as request

xxii. Line 111, authors used forward variable selection method when constructing logistic regression model. What was the justification for choosing this method as oppose to other methods? Further, in forward selection inclusion of a new variable may make an existing variable in the model non-significant. How did the authors dealt with this drawback? If so how?

Thank you for your comment. Logistic regression is a classification algorithm used to find the probability of event success and event failure. It is used when the dependent variable is binary(0/1, True/False, Yes/No) in nature. It is suitable for the purpose of developing our model to predict DILI occurrence using ramipril.

In our study, we deal with negative confounders by using multivariate analysis. Multivariate models can handle large numbers of covariates (and also confounders) simultaneously. For example in our study that aimed to measure the relation between 23 variables in the same model. Logistic regression is considered to provide a better fit to the data if it demonstrates an improvement over a model with fewer predictors.

xxiii. In 128, the use of abbreviation H0 should first be written in full before using it even if it is a well- known abbreviation.

Thank you for your comment. We have revised our manuscript as request

xxiv. Line 138, indicates that ethical approval was obtained from the ethics subcommittees of the Hai Phong International Hospital. Authors need to provide evidence by including the reference number.

Thank you for your comment. We have revised our manuscript as request
xxv. In this study, authors have claimed that rimipril caused DILI. It is important to recognize that causality cannot be established definitively through a retrospective cohort study which is an observation study; however, such studies are powerful tool that can provide important evidence to suggest causality, as well as information regarding the strength of an association between ramipril and DILI. What authors have reported is an association between ramipril and DILI.

We totally agree with your opinion. Our study provided for the first time an association between ramipril use and DILI. Other studies described only individual cases. We tried to reduced the bias of drug exposure. The identification of the cause of DILI was determined not only by the patient's memory but also by the carefully maintained medical records.. The limitations of the study were also added to the discussion with the following content:

This case-control study tested a hypothesis about the correlation between intake of ramipril and DILI. Admittedly, our study is not as powerful as the other studies with a different study design in terms of establishing a causal relationship. Nevertheless, our study provided data and a reference for future clinical studies that would be using more rigorous scientific methods. Moreover, identification of the cause of DILI may be influenced by certain drugs or supplements that cause DILI, but to the best of our knowledge, this has not been reported in the literature.

xxvi. It is important for authors to give a biological plausibility to establish the observed relationship and identify a scientifically valid mechanism by which the ramipril can associated with DILI.

Thank you for your comments. We showed the scientifically mechanism and evidence from literature about relationship of ramipril and DILI in introduction and discussion sections as below:

However, only a few cases of ramipril resulting in liver injury have been documented, but the rare instances that have been published have resembled typical ACE inhibitor-related acute liver injury (9, 10). Previous studies have demonstrated the hepatotoxic potential of ramipril, highlighting the need for physicians to be vigilant about this problem (10).

Until now there are five case reports of ramipril-induced DILI (9, 10, 18).

xxvii. Surprisingly, authors have not included limitations in this study and the findings of this study could be distorted by many factors not limited to biases, misclassification and measurement error, confounding and effect modification (also called “interaction”).

The limitations of the study were also added to the discussion with the following content:

This case-control study tested a hypothesis about the correlation between intake of ramipril and DILI. Admittedly, our study is not as powerful as the other studies with a different study design in terms of establishing a causal relationship. Nevertheless, our study provided data and a reference for future clinical studies that would be using more rigorous scientific methods. Moreover, identification of the cause of DILI may be influenced by certain drugs or supplements that cause DILI, but to the best of our knowledge, this has not been reported in the literature.

xxviii. In line 147, under the results for table 1, the footnotes are not complete.

Thank you for your comment. We have revised our manuscript as request
xxix. In line 151, authors have reported that “average age of 64.2, and the majority were men (57%)”. Authors should explain the central measure of tendency used with units and why? Further, the reported percentage should be accompanied by an absolute number.

Thank you for your comment. We have revised our manuscript as request

xxx. In line 154, there is a statement that “on the day of admission, the ALT test results of the ramipril group averaged 31.8 74.6 (UI 155 / L)” This should be made clearer to the readership. This applies to a number of sections where such results have been reported.

Thank you for your comment. We have revised our manuscript as below:

On the day of prescription medication causing DILI, the ALT test results of the ramipril group averaged 31.8 ±74.6 (UI / L), similar to the group using other ACEIs 16.1± 18.1 with p=0.11.

xxxi. In a number of sections such lines 158 and 159, authors have not indicated the reported central measure of tendency. This should be made clear.

Thank you for your comment. We have revised our manuscript
xxxii. In table 2, authors have reported (n±SD) suggesting number and standard deviation for age and length of hospital stay. This is not appropriate. Standard deviation should be reported with appropriate measure of central tendency. This applies to the other tables in the results section.

Thank you for your comment. We have revised with mean as central tendency.
xxxiii. What statistical tests were performed to obtain the p-values for different variables in table 2 and other tables in the results?

Thank you for your comment. We added required information in statistic analysis section
xxxiv. Although there are some footnotes under table 2, there are some still missing. This applies to table 3a.

Thank you for your comment. We have revised our manuscript
xxxv. What were the comorbidity in the study?

Thank you for your comment. In our study, we did not describe any type of comorbidities. We only counted the number of comorbidities diagnosed according to the ICD-10 disease code

xxxvi. The authors used multivariate logistic regression model. Was this really multivariate logistic regression or multivariable regression?

Thank you for question. We used the method of logistic regression for developing a classification algorithm used to find the probability of DILI.

xxxvii. In the discussion the authors keep repeating the estimate (odds ratio) and p-value. This is repetition of results. Authors should just discuss!

Thank you for your comment. We have revised our manuscript as your suggestion.

xxxviii. In line 245, there is a statement that “on the day of DILI, the ALT and ALP levels in our patient were elevated by 8.2” Which particular patient is being referred to and why?

Thank you for your comment. We have revised our manuscript as below:

On the day of DILI, the ALT and ALP levels in patients using ramipril were elevated by 8.2-and 14.1-fold, respectively

xxxix. Authors should make it clear whether which method of analysis they used. Either intention to treat analogue or per protocol analysis and why?

Thank you for your question. To make the method of statistic analyis clearer, we revised this section as below:

The logistic regression model was built using the R statistical software, version 3.2.4 (A Language and Environment for Statistical Computing, Vienna, Austria (14). We used forward selection based on the chi-square test of the change in residual deviance. Differences among groups were analyzed using the chi-square test for qualitative variables, one-way analysis of variance for continuous variables with normal distributions, and the non-parametric Kruskal‐Wallis test for continuous variables with non-standard distributions. We used a cutoff value of P<0.001 to add new terms.

xl. Authors have recommended that clinicians should be aware of age and liver disease before prescribing ramipril to avoid the risk of DILI in patients. This is surprising since age showed no difference between DILI and ACEIs groups as well as liver disease at 97.5% CI. What was the rational for this recommendation?

Thank you for this review. In Table 1, we only compared the median ages in the ramipril group and other comparable ACEIs, but we did not compare liver disease. However, liver disease was a significant risk factor when we compared groups with and without DILI in the ramipril-using population (Tables 3a,b,c). When building a logistic linear regression model, we find the prevalence of DILI in the group of subjects using ramipril (1687 with non_DILI and 117 subjects with DILI) (Figure 1)

**Reviewer #2: A well written paper on an important subject. The following corrections should be made.**

Line 65, the word first is out of place, one expects it to be followed by a second action, which is not done in this sentence.

Thank you for your comment. We have revised our manuscript

Line 72-26 - Subjects
This sentence belongs to the ethics section, not where it is currently.

Thank you for your comment. We have revised our manuscript as your suggestion

Line 154-156 - the ± sign is missing from the numbers in the text and needs to be corrected.

Thank you for your comment. We have revised our manuscript as your suggestion
Analysis
Sex and duration/how long someone has been on treatment, although found to be important factors in other studies, have not been included in the model or discussed at all. This needs to be explained.

Thank you for your comment. Gender characteristics and duration of drug use have been added and described in Table 2 and discussed in the Discussion section

Discussion
A discussion of factors associated with DILI from Ramipril (from the modeling) needs to be placed within the context of findings from similar studies in the literature. This has not been done. Are your findings novel, similar or different from those found by similar studies?

Our study provided for the first time an association between ramipril use and DILI. Other studies described only individual cases. This described in introduction and discussion sections as below:

Thank you for your comment

However, only a few cases of ramipril resulting in liver injury have been documented, but the rare instances that have been published have resembled typical ACE inhibitor-related acute liver injury (9, 10). Previous studies have demonstrated the hepatotoxic potential of ramipril, highlighting the need for physicians to be vigilant about this problem (10).

Until now there are five case reports of ramipril-induced DILI (9, 10, 18).

Limitations
Authors need to discuss the limitations of their findings. Why should we believe these findings?

Thank you for your comment

The limitations of the study were also added to the discussion with the following content:

This case-control study tested a hypothesis about the correlation between intake of ramipril and DILI. Admittedly, our study is not as powerful as the other studies with a different study design in terms of establishing a causal relationship. Nevertheless, our study provided data and a reference for future clinical studies that would be using more rigorous scientific methods. Moreover, identification of the cause of DILI may be influenced by certain drugs or supplements that cause DILI, but to the best of our knowledge, this has not been reported in the literature.

**REFERENCE**

1.	Danan G, Teschke R. Drug-induced liver injury: why is the Roussel Uclaf Causality Assessment Method (RUCAM) still used 25 years after its launch? Drug safety. 2018;41(8):735-43.

2.	Teschke R, Wolff A. Regulatory causality evaluation methods applied in kava hepatotoxicity: are they appropriate? Regulatory Toxicology and Pharmacology. 2011;59(1):1-7.

3.	Danan G, Benichou C. Causality assessment of adverse reactions to drugs--I. A novel method based on the conclusions of international consensus meetings: application to drug-induced liver injuries. J Clin Epidemiol. 1993;46(11):1323-30.
