# Supplementary material for: A logistic regression model based on inpatient health records to predict drug-induced liver injury caused by ramipril—An angiotensin-converting enzyme inhibitor
Source: PLoS One. 2022 Aug 17;17(8):e0272786. doi: 10.1371/journal.pone.0272786 (PMC9384991; doi:10.1371/journal.pone.0272786)
Supplement: Attachment — Submitted filename: Response to Reviewer_2.docx [file pone.0272786.s003.docx]

Dear editor and reviewers,

Thank you for giving us the opportunity to submit a revised draft of the manuscript “**A logistic regression model based on inpatient health records to predict drug-induced liver injury caused by ramipril – an angiotensin-converting enzyme inhibitor**” for publication in *PLOS ONE*. We appreciate the time and effort that you and the reviewers dedicated to providing feedback on our manuscript and are grateful for the insightful comments on and valuable improvements to our paper. We have incorporated most of the suggestions made by the reviewers. Please see below, editor/reviewer comments are black and our responses are in blue.

**Editor comments**

PLOS ONE's publication criteria require that the data presented in PLOS ONE manuscripts must support the conclusions drawn (<http://journals.plos.org/plosone/s/criteria-for-publication#loc-4>). We have some concerns about the conclusions in the manuscript. This is a retrospective observational study. However, here there are claims related to causation, e.g. "Our study demonstrated that using ramipril increases the risk of DILI" and "We found that taking ramipril increased the 2.68-fold risk of DILI (OR=2.68, p<0.001) compared to other ACEIs". Please revise your manuscript to address any instances of claims that are not supported by your results, e.g lines 251-252, lines 259-260 and lines 266-268 and also address the limitations of your study.

Thank you for your comment. We revised our manuscript based on your suggestion.

Our study demonstrated that using ramipril increases the risk of DILI → *From our study we have evidence that the odds of developing DILI is around two and a half times higher for ramipril using group compared non-use of ramipril*

On the day of DILI, the ALT and ALP levels in patients who took ramipril were elevated by 8.2-and 14.1-fold, respectively → *Taking ramipril group was observed to be higher risk of developing DILI compared to other ACEIs*

The limitation was revised as below:

This case-control study tested a hypothesis about the correlation between intake of ramipril and DILI. Admittedly, our study is not as powerful as the other studies with a different study design in terms of establishing a causal relationship. Nevertheless, our study provided data and a reference for future clinical studies that would be using more rigorous scientific methods. Another limitation of our study is the patient's adherence to medication because our study subjects are outpatients. However, to measure adherence to drug therapy, we used the patient’s self report in every hospital visit. Moreover, identification of the cause of DILI may be influenced by certain drugs or supplements that cause DILI, but to the best of our knowledge, this has not been reported in the literature.
