# Supplementary material for: A logistic regression model based on inpatient health records to predict drug-induced liver injury caused by ramipril—An angiotensin-converting enzyme inhibitor
Source: PLoS One. 2022 Aug 17;17(8):e0272786. doi: 10.1371/journal.pone.0272786 (PMC9384991; doi:10.1371/journal.pone.0272786)
Supplement: Attachment — Submitted filename: PONE-D-21-13763_R2_reviewer05282022.docx [file pone.0272786.s004.docx]

PONE-D-21-13763_R2_reviewer05282022

This study is about establishing approaches to predict the ramipril induced liver injury. Authors selected 1686 inpatients to generate 3 models predicting DILI by using logistic regression. But the processing of building risk estimation models is not clear, what are the criteria to keep those variables in model 1, 2, 3, respectively. Authors stated they used 2-fold cross-validation technique to evaluate the predictive performance, but the validation evaluation results with a1 and a2 were not presented. When doing the performance measurement by using ROC curve, authors did not provide the risk scores and corresponding cutoff points of each model. Based on the ROC curves, three models have perfect performances of predicting IDLI, but it was too good to be true because all three models can nearly predict every DILI case with very small part of false positive. If this paper just only wanted to identify the risk factors for ramipril induced liver injure prevention, the odd ratio (OR) of multiple regression is enough for drawing reliable conclusions.

Furthermore, Table 2 is not necessary because it is not relevant to the study purposes. Instead, characteristics of included patients (1686) should be presented in results part.
