# Supplementary material for: A logistic regression model based on inpatient health records to predict drug-induced liver injury caused by ramipril—An angiotensin-converting enzyme inhibitor
Source: PLoS One. 2022 Aug 17;17(8):e0272786. doi: 10.1371/journal.pone.0272786 (PMC9384991; doi:10.1371/journal.pone.0272786)
Supplement: Attachment — Submitted filename: response_to_reviewer_3.docx [file pone.0272786.s005.docx]

Dear editor and reviewers,

Thank you for giving us the opportunity to submit a revised draft of the manuscript “**A logistic regression model based on inpatient health records to predict drug-induced liver injury caused by ramipril – an angiotensin-converting enzyme inhibitor**” for publication in *PLOS ONE*. We appreciate the time and effort that you and the reviewers dedicated to providing feedback on our manuscript and are grateful for the insightful comments on and valuable improvements to our paper. We have incorporated most of the suggestions made by the reviewers. Please see below, editor/reviewer comments are black and our responses are in blue.

**Reviewer comments**

This study is about establishing approaches to predict the ramipril induced liver injury. Authors selected 1686 inpatients to generate 3 models predicting DILI by using logistic regression. But the processing of building risk estimation models is not clear, what are the criteria to keep those variables in model 1, 2, 3, respectively. Authors stated they used 2-fold cross-validation technique to evaluate the predictive performance, but the validation evaluation results with a1 and a2 were not presented. When doing the performance measurement by using ROC curve, authors did not provide the risk scores and corresponding cut off points of each model. Based on the ROC curves, three models have perfect performances of predicting IDLI, but it was too good to be true because all three models can nearly predict every DILI case with very small part of false positive. If this paper just only wanted to identify the risk factors for ramipril induced liver injure prevention, the odd ratio (OR) of multiple regression is enough for drawing reliable conclusions.
Furthermore, Table 2 is not necessary because it is not relevant to the study purposes. Instead, characteristics of included patients (1686) should be presented in results part.

Q1: This study is about establishing approaches to predict the ramipril induced liver injury. Authors selected 1686 inpatients to generate 3 models predicting DILI by using logistic regression. But the processing of building risk estimation models is not clear, what are the criteria to keep those variables in model 1, 2, 3, respectively.

Response:

Thank you for your comments. We have revised the Methods section of our manuscript as follows:

The logistic regression model was built using the R statistical software, version 3.2.4 (A Language and Environment for Statistical Computing, Vienna, Austria) (14). To develop the DILI risk estimation model, we mapped the variables collected by the MHR to 23 discrete variables.  The criteria for the inclusion of a variable in our model were based on clinical and statistical significance. We used forward selection based on the chi-square test of the change in residual deviance. Our goal was to build a logistic regression model that minimizes the variables until the optimal model that describes the data is found. We constructed three risk estimation models. These models used the presence or absence of DILI as the dependent variable and the 23 aforementioned variables as independent variables. We used a cutoff value of P<0.001 to add new terms. Differences among groups were analyzed using the chi-square test for qualitative variables, one-way analysis of variance for continuous variables with normal distributions, and the non-parametric Kruskal–Wallis test for continuous variables with non-standard distributions.

Q2: Authors stated they used 2-fold cross-validation technique to evaluate the predictive performance, but the validation evaluation results with a1 and a2 were not presented.

Response:

Thank you for your comment.

We created multiple random splits of the dataset and divided them into training and validation datasets. For each split, the model was fitted to the training data, and predictive accuracy was assessed using the two-cross validation method. The results were then averaged over the splits.

The model performance is presented in Table 4 including specificity, sensitivity, and A_ROC_

Q3: When doing the performance measurement by using ROC curve, authors did not provide the risk scores and corresponding cutoff points of each model.

Response:

Thank you for your suggestion.

The Youden index method was used to find the optimal cut-off point, which is the difference between the number of true positives and the number of false positives over all possible cut-off point values. I would like to add the cut-off point in each model in Table 4 as follows:

| **Model** | **Cutoff point** | **Specificity** | **Sensitivity** | **Accuracy** | **P-value^#^** | **A_ROC_^!^** | **95% CI^$^** |
| --- | --- | --- | --- | --- | --- | --- | --- |
| Model 1 | 0.024 | 96.08 | 94.34 | 95.97 |  | 0.993 | 0.987 – 0.998 |
| Model 2 | 0.026 | 96.58 | 98.11 | 96.68 | 0.37 | 0.995 | 0.991 – 0.999 |
| Model 3 | 0.023 | 96.46 | 92.45 | 95.64 | 0.002 | 0.992 | 0.986 – 0.999 |
| #: Anova test compared to model 1  A_ROC_: Area under the receiver operating characteristic curve; CI: confidence interval | | | | | | | |

Q4: Based on the ROC curves, three models have perfect performances of predicting IDLI, but it was too good to be true because all three models can nearly predict every DILI case with very small part of false positive. If this paper just only wanted to identify the risk factors for ramipril induced liver injure prevention, the odd ratio (OR) of multiple regression is enough for drawing reliable conclusions.

Response:

Thank you for your valuable comments. We strongly agree that OR is sufficient to explore the risk factors for ramipril-induced DILI. However, we also wanted to build a model that includes all significant variables to predict the risk of DILI caused by ramipril, supporting decision-making in clinical practice.

Q5: Furthermore, Table 2 is not necessary because it is not relevant to the study purposes. Instead, characteristics of included patients (1686) should be presented in results part.

Response:

Thank you for comments. We would like to add the characteristics of all patients (1686 patients) to Table 1. We would like to retain table 2 because it supports the selection of variables in the model.

Table 1: Characteristics of study subjects (n=1686)

| **Clinical data** | **Value** |
| --- | --- |
| Age, years (mean ± SD) | 66.48±10.04 |
| Weight, kg (mean ± SD) | 45±12.4 |
| Male (n (%)) | 999 (59.2) |
| BMI (mean ± SD) | 22.41±4.29 |
| Length of stay, days (mean ± SD) | 14 ±4.5 |
| Duration of taking drug, days (mean ± SD) | 30.1± 8.7 |
| Laboratory characteristics (mean ± SD) |  |
| ALT, U/l | 37.9±67.1 |
| AST, U/l | 37.5±92.4 |
| ALP, U/l | 37.1±70.7 |
| TBiL, µmol/l | 1.3±5.5 |
