# Supplementary material for: A logistic regression model based on inpatient health records to predict drug-induced liver injury caused by ramipril—An angiotensin-converting enzyme inhibitor
Source: PLoS One. 2022 Aug 17;17(8):e0272786. doi: 10.1371/journal.pone.0272786 (PMC9384991; doi:10.1371/journal.pone.0272786)
Supplement: Attachment — Submitted filename: Response to Reviewer_4.docx [file pone.0272786.s006.docx]

Dear editor and reviewers,

Thank you for giving us the opportunity to submit a revised draft of the manuscript “**A logistic regression model based on inpatient health records to predict drug-induced liver injury caused by ramipril – an angiotensin-converting enzyme inhibitor**” for publication in *PLOS ONE*. We appreciate the time and effort that you and the reviewers dedicated to providing feedback on our manuscript and are grateful for the insightful comments on and valuable improvements to our paper. We have incorporated most of the suggestions made by the reviewers. Please see below, editor/reviewer comments are black and our responses are in blue.

**Editor comments**

Reviewer #3: The revised paper added more details and solved some of my concerns. But still I have little confidence about the reliability of those three models, because they are too good to be true (I can’t believe it!). Authors, based on their models, created 3 new indexes. We can call these indexes as risk score and authors this time provided the optimal cutoff points of those 3 risk scores for predicting of DILI. By using these risk scores, we then could perfectly predict the DILI. It is out of common sense because the components of those risk scores have weak or not strong impacts on DILI with margin positive OR values (in table 4). Therefore, in order to justify the performance of those models, author should double check their analysis and provide the details of those 3 risk scores in their studying population.

We thank you for your thoughtful suggestions and insights. The manuscript has benefited from these insightful suggestions.

All steps of data analysis, model construction and validation were rechecked by our researchers. We admit that there was an error in the A_ROC_ calculation due to using the wrong data set in R software. We have checked and corrected it according to your suggestion. Also, we have added the Fig 4 to better describe the details of our model.

Table 5. Comparison of the performances and the area under the ROC curves of the models.

| **Model** | **Cutoff point** | **Specificity** | **Sensitivity** | **P-value^#^** | **A_ROC_^!^** | **95% CI^$^** | **AIC** | **R^2^** |
| --- | --- | --- | --- | --- | --- | --- | --- | --- |
| Model 1 | 0.029 | 0.884 | 0.727 |  | 0.831 | 0.766-0.897 | 147.17 | 0.375 |
| Model 2 | 0.024 | 0.865 | 0.727 | 0.32 | 0.825 | 0.758-0.892 | 143.69 | 0.366 |
| Model 3 | 0.033 | 0.896 | 0.682 | 0.29 | 0.820 | 0.752-0.888 | 142.8 | 0.372 |

Figure 4. Comparison of receiver operating characteristic curve curves of 3 models. AUC: area under the receiver operating characteristic curve

The limitation of our study was revised as below:

This case-control study tested a hypothesis about the correlation between intake of ramipril and DILI. Admittedly, our study is not as powerful as the other studies with a different study design in terms of establishing a causal relationship. Nevertheless, our study provided data and a reference for future clinical studies that would be using more rigorous scientific methods. Another limitation of our study is the patient's adherence to medication because our study subjects are outpatients. However, to measure adherence to drug therapy, we used the patient’s self-report in every hospital visit. Moreover, identification of the cause of DILI may be influenced by certain drugs or supplements that cause DILI, but to the best of our knowledge, this has not been reported in the literature. Although the independent variables were statistically significant related to DILI appreance our model performance had a low R-squared value. The R2 value is an overall measure of strength of association between the model and the response and does not reflect the contribution of independent predictor variable.
